# 

**Published:** 1881-08

**Authors:** 


### AMERICAN SOCIETY OF MICROSCOPISTS.

The Third Annual Session of the American Society of Microscopists will be held in Columbus, O., in the rooms of the Board of Trade, City Hall Building, beginning on Tuesday, August 9th, and continuing three days.

I am authorized by the Tyndall Association of Natural Sciences to extend in their behalf a cordial invitation to Columbus to all members of the Society, present or prospective.

An endeavor will be made to provide entertainment in private families for all who desire it. All such are requested to send their names at as early a date as possible to Rev. I. F. Stidham, Columbus, Ohio, Chairman of the Committee of Arrangements who will also furnish members with any desired information.

Duplicates of the enclosed blanks can be obtained of the Treasurer and Custodian, Mr. Geo. E. Fell, Buffalo, N. Y.

ALBERT H. TUTTLE, Secretary.

A MONTHLY MAGAZINE DEVOTED TO THE INTERESTS OF

**THE DENTAL PROFESSION.**

*TERMS REDUCED TO $2.50 PER YEAR. SINGLE COPIES 25C.*

EDITED BY PROF. J. TAFT, D.D.S.

AND

*Published by SPENCER, CROCKER & CO., Ohio Dental Depot.*

The volume commences in January and closes in December.

The REGISTER being issued monthly is always fresh, therefore Indispensable to the practitioner who desires to keep posted up with the new inventions and improvements which are daily developed. Neither expense nor effort will be spared to give value and variety to its contents. Articles will be illustrated with well executed engravings whenever they will add to the interest or assist to explanation.

Its extensive circulation and frequency of issue make it one of the BEST DENTAL ADVERTISING MEDIUMS in the United States.

All subscriptions must begin with January or July.

Local or special notices, five lines or less, will be inserted for $3 each insertion; over five lines, 50 cts. per line.

All advertising bills payable in advance, or at furthest, after the issue of the first number containing the advertisement. All transient advertisements to be paid for in advance.

☞CONTRIBUTIONS SOLICITED.

Exclusively Editorial Communications should be addressed to Editor.

The latest number of the REGISTER may be ordered with or without other goods at any time, and all letters should be addressed to

